# Giant symptomatic adrenal myelolipoma: A case report

**DOI:** 10.1016/j.amsu.2022.103333

**Published:** 2022-02-09

**Authors:** Abdelbassir Ramdani, Asmae Aissaoui, Tariq Bouhout, Amal Bennani, Hanane Latrech, Badr Serji, Tijani El Harroudi

**Affiliations:** a Surgical Oncology Department, Mohammed VI University Hospital, Regional Oncology Center, Oujda, Morocco; b Mohammed First University Oujda, Faculty of Medicine and Pharmacy Oujda, Oujda, Morocco; c Pathology Department, Mohammed VI University Hospital, Oujda, Morocco; d Endocrinology Department, Mohammed VI University Hospital, Oujda, Morocco

**Keywords:** Adrenal myelolipoma, Case report, Endocrine surgery

## Abstract

**Introduction:**

Adrenal myelolipomas are rare non-functioning benign tumors composed of adipose and hematopoietic tissues. Most AMLs are discovered incidentally and represent the second most common adrenal incidentaloma.

**Case presentation:**

A 58-years-old female patient, obese with a history of diabetes and blood hypertension presented with complaints of pain in the left flank. Abdominopelvic computed tomography showed a giant well-defined mass of the left adrenal gland with fat density suggesting adrenal myelolipoma. The patient underwent open left adrenalectomy. The pathological study confirmed the diagnosis of adrenal myelolipoma.

**Discussion:**

Most AMLs are asymptomatic, remain stable in size, or grow slowly. Mass effect symptoms and spontaneous rupture are observed more in larger AMLs. The most common symptoms observed are abdominal discomfort/pain, hypochondrial pain, and flank pain. Most of the AMLs are discovered incidentally and the radiological features are accurate in diagnosing AML in up to 90% of the cases, CT is more sensitive for detection than other imaging modalities. The open surgery approach is the standard treatment of choice for giant AML (>10cm) while the minimally invasive approach has been used in only a few cases.

**Conclusion:**

The therapeutic management is discussed on a case-by-case basis. Surgical treatment is indicated for larger, symptomatic, or rapidly growing AMLs. Meanwhile smaller and asymptomatic AMLs are managed conservatively.

## Highlights

- Adrenal myelolipoma are rare benign tumors composed of adipose and hematopoietic tissues.
- Most adrenal myelolipoma are asymptomatic and discovered incidentally.
- Surgical treatement is indicated for larger, symptomatic or rapidly growing adrenal myelipoma.
- Smaller and asymtomatic adrenal myelolipoma are managed conservatively.

## 1 Introduction

Adrenal myelolipomas (AMLs) are rare non-functioning benign tumors composed of adipose and hematopoietic tissues [1]. AMLs are found in one out of 500–1250 autopsy cases [2]. However, the exact clinical prevalence of the tumor is impossible to assess because of the high percentage of asymptomatic cases and its benign nature [2]. Most AMLs are asymptomatic and discovered incidentally; they represent the second most common adrenal incidentaloma with 6–16% of all incidental adrenal masses [1,2]. The Radiological features are specific and suggest the diagnosis in more than 90% of the cases [3]. Myelolipoma is defined as ‘giant’ when its greatest diameter is > 10 cm [4]. Giant AMLs are exceptional and reported only in a few studies Herein, we report a rare case of a giant symptomatic left AML in a female patient. This case has been reported following the SCARE criteria [5].

## 2 Case report

**Fig. 1:**
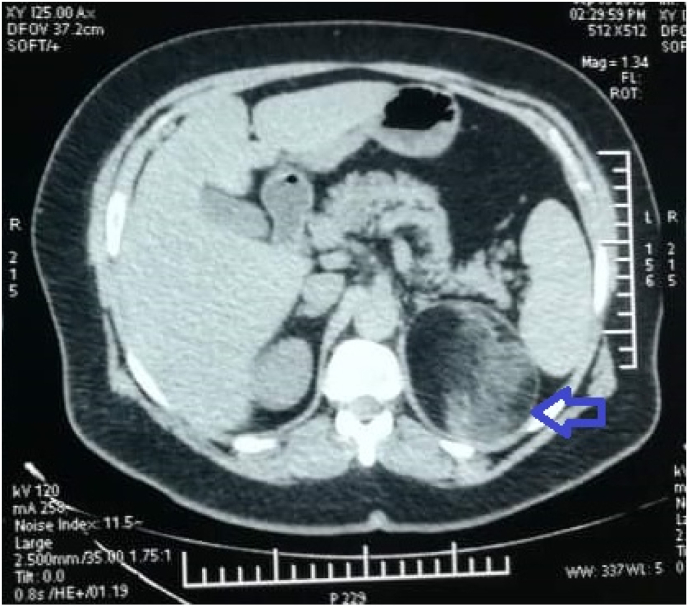
Abdominal computed tomography scan showing a large well-defined mass of the left adrenal gland with fat density suggesting myelolipoma (Blue arrow).

A 58-years-old female patient, obese (BMI: 35Kg/m^2^) with a history of diabetes and blood hypertension under treatment with no endocrine disorders or associated comorbidities in the patient and relatives, presented with a three months history of vague, dull pain in the left flank without fever or urinary signs. Physical examination revealed a slight tenderness in the left lumbar region without any palpable mass. Abdominal ultrasound showed the presence of a hyperechoic mass in the left suprarenal region with undefined margins. An abdominopelvic computed tomography (CT) showed a large well-defined mass of the left adrenal gland measuring 111 × 82 × 82 mm with fat density (−80 UH) suggesting an adrenal myelolipoma (Fig. 1). Laboratory tests, including routine blood examination, serum cortisol 8 a.m., 24-h urine cortisol excretion, Urinary normetanephrine, and metanephrine, were all within the normal range.

**Fig. 2:**
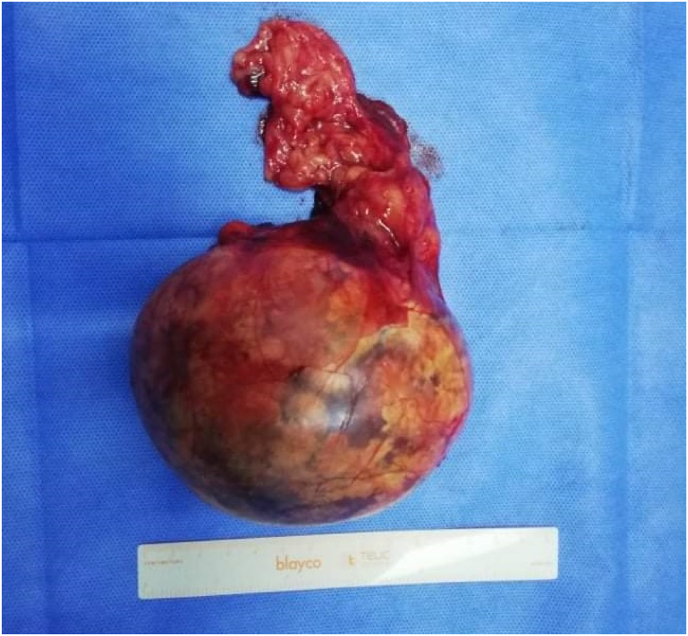
Image showing the specimen.

**Fig. 3:**
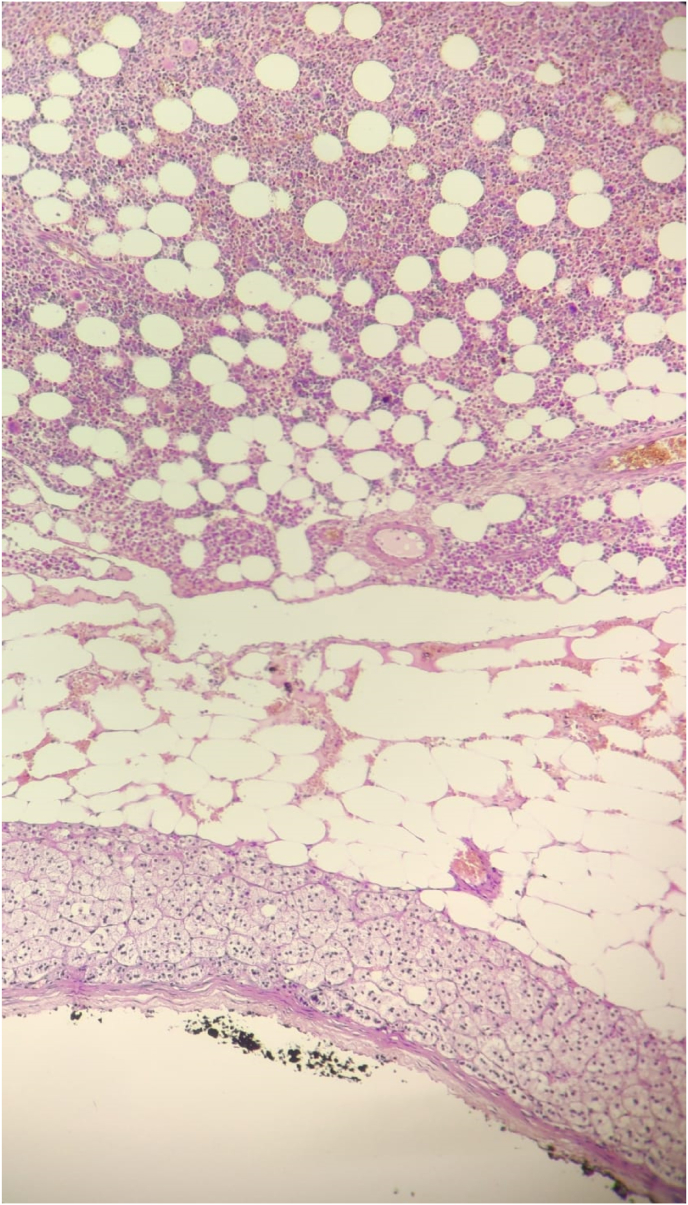
Microphotograph showing mature adipocyte mixed with hematopoietic tissue.

The patient underwent open left adrenalectomy. The specimen size was 11 × 13 × 6 cm and weighed 400 g (Fig. 2); Pathology examination showed the presence of fat and hematopoietic tissues confirming the diagnosis of adrenal myelolipoma (Fig. 3). The patient made an uneventful recovery and was discharged from the hospital on postoperative day five. She remained asymptomatic at a one-year follow-up.

## 3 Discussion

Described for the first time by Gierke in 1905 [6]. AML tends to occur more frequently in the fifth and sixth decades with a mean age of 51 years without any gender predominance [2]. According to a large study, 73% of the patient suffered from hypertension and more than half of the patients were obese with BMI >30kg/m^2^ [7]. According to Decmann et al., 59.2% of the AML reported in their review were located in the right adrenal gland and only 12.3% were bilateral [2]. In a large study conducted by Hamidi et al., only 11 patients among 305 had AML ≥10 cm [1]; the largest reported AML in the literature was measuring 31 cm × 24.5 cm × 11.5 cm and weighing 6 kg [8]. Myelolipomas remain stable in size or grow slowly, in a large longitudinal follow-up study, overall tumor change ranged from −10 mm to 115 mm with growth rate ranging from −6 mm/year to 14 mm/year [1].

Most AMLs are asymptomatic, mass effect symptoms and spontaneous rupture are observed more in AML >6cm [1,9]. The most common symptoms observed are abdominal discomfort/pain, hypochondrial pain, and flank pain [2]. Most of the AMLs are discovered incidentally and the radiological features are accurate in diagnosing AML in up to 90% of the cases [9], ultrasound is not accurate and may show hypoechoic or hyperechoic mass depending on the predominance of fat or myeloid tissue [10]. CT is more sensitive for detection than other imaging modalities [11], AMLs appear well-defined, hypodense, and heterogeneous masses; the presence of fat density is essential for the radiological diagnosis of AML [2]. On magnetic resonance imaging (MRI), fat tissue demonstrates high-intensity signal on T1-weighted sequence and loss of signal intensity in fat suppression T1-weighted sequence, this feature confirms the diagnosis [12]. The fat compound of AMLs is the key factor for preoperative diagnosis on CT or MRI [13]. According to The AACE/AAES Guideline on Adrenal Incidentaloma (2009) AMLs are an exception to the mandatory endocrine/metabolic workup [14]. However, a review of the literature shows that a significant number of AMLs are either secreting hormones or occur in association with either CAH or ACTs. Avoiding metabolic workup may result in missing out on these conditions [8]. Few studies reported patients with giant AML (>10cm) [15,16]. 12 cases were reported in two different studies conducted by Hsu et al. [14] & Gadelkareem et al. [16]. Table 1 summarizes the demographic and clinical characteristics of the giant AMLs in comparison with our case report.

**Table 1:** Demographic and clinical characteristics of the patients with giant adrenal myelolipoma.

Patient	Gender	Age	Presentation	BMI (Kg/m^2^)	Imaging	Side	Tumor size (cm)	HU value
1 (Hsu et al.)	F	44	Incidentaloma	*	CT/MRI	Right	14	*
2 (Hsu et al.)	M	48	Left palpable mass	*	CT	Left	13.5	*
3 (Hsu et al.)	M	45	Abdominal pain	*	CT/MRI	Right	16.5	*
4 (Gadelkareem et al.)	M	56	Incidentaloma	29.39	CT	*	10	−30 to −40
5 (Gadelkareem et al.)	F	48	Loin pain	27.44	CT	*	12	−20 to −50
6 (Gadelkareem et al.)	F	62	Incidentaloma	29.41	CT	*	11.5	−25 to −40
7 (Gadelkareem et al.)	F	63	Incidentaloma	29.33	CT	*	14	−25 to 50
8(Gadelkareem et al.)	F	44	Incidentaloma	30.45	CT	*	15	−25 to −35
9 (Gadelkareem et al.)	F	33	Loin pain	37.37	CT	*	13	−20 to −35
10 (Gadelkareem et al.)	F	45	Incidentaloma	29.64	CT	*	10	−20 to −35
11 (Gadelkareem et al.)	M	45	Incidentaloma	34.64	CT/MRI	*	16	−20 to −30
12 (Gadelkareem et al.)	F	47	Loin pain	28.6	CT	*	12	−15 to 30
Our patient	F	58	Loin pain	35	CT	Left	13	−80

Differential diagnoses include retroperitoneal lipoma, retroperitoneal liposarcoma, retroperitoneal leiomyosarcoma, extra-renal angiomyolipoma, and primary or metastatic adrenal malignancy [15]. Management of adrenal myelolipoma should be per individual basis. Smaller (<4cm) and asymptomatic AML can be managed conservatively and patients are monitored by abdominal CT or MRI annually or biannually [17], meanwhile, surgical treatment is reserved for symptomatic, larger (>6cm) or rapidly growing AML; suspicion of malignancy constitutes another indication for surgical resection [[17], [18], [19]]. The open surgery approach is the standard treatment of choice for giant AML (>10cm) while the minimally invasive approach has been used in only a few cases [17,20]. In our case, open left adrenalectomy was performed as the tumor was giant and symptomatic. Table 2 illustrates the management of giant AMLs with the outcome and follow-up.

**Table 2:** *Management, outcome and follow up of patients with giant adrenal myelolipoma*.

Patient	Operative technique	Hospital stay	Operative time	Convalescence	Follow up
1 (Hsu et al.)	Open (Reversed L incision)	*	*	*	33 months
2 (Hsu et al.)	Open (Midline incision)	*	*	*	114 months
3 (Hsu et al.)	Open (subcostal incision)	*	*	*	25 months
4 (Gadelkareem et al.)	Open (thoracolumbar)	7 days	220 min	Uneventful	*
5 (Gadelkareem et al.)	Open (Subcostal incision)	8 days	200 min	Uneventful	*
6 (Gadelkareem et al.)	Open (Subcostal incision)	6 days	225 min	Intestinal obstruction	*
7 (Gadelkareem et al.)	Open (Thoracolumbar)	6 days	180 min	Uneventful	*
8(Gadelkareem et al.)	Open (Thoracolumbar)	9 days	205 min	Uneventful	*
9 (Gadelkareem et al.)	Open (Subcostal incision)	6 days	175 min	Uneventful	*
10 (Gadelkareem et al.)	Laparoscopy	5 days	225 min	Uneventful	*
11 (Gadelkareem et al.)	Laparoscopy	3 days	225 min	Uneventful	*
12 (Gadelkareem et al.)	Open (Subcostal incision)	7 days	210 min	Uneventful	*
Our patient	Open (Midline incision)	5 days	180 min	Uneventful	12 months

## 4 Conclusion

Most AMLs are asymptomatic and discovered incidentally. The radiological findings are accurate and make the diagnosis in more than 90% of cases. The therapeutic management is discussed on a case-by-case basis. Surgical treatment is indicated for larger, symptomatic, or rapidly growing AMLs meanwhile smaller and asymptomatic AMLs are managed conservatively.

## Ethics approval

No ethical approval necessary.

## Source of funding

The author(s) received no financial support for the research, authorship and/or publication of this article.

## Author contributions

**Ramdani Abdelbassir:** Writing, review and editing of the manuscript.

**Asmae Aissaoui, Amal Bennani**: Provided the pathological analysis.

**Bouhout Tariq, Latrach Hanane, Serji Badr:** Contributed for diagnose and treatment of the patient.

**El Harroudi Tijani:** Supervised the writing of manuscript.

## Registration of research studies

Our paper is a case report; no registration was done for it.

## Guarantor

Ramdani Abdelbassir.

## Consent of the patient

Written informed consent was obtained from the patient for publication of this case report and accompanying images. A copy of the written consent is available for review by the Editor-in-Chief of this journal on request.

## Provenance and peer review

Not commissioned, externally peer reviewed.

## Declaration of competing interest

The authors declared no potential conflicts of interests with respect to research, authorship and/or publication of the article.
